# Therapeutic baths with glyphosate have antiparasitic effects on monogeneans in the gills of *Colossoma macropomum* (tambaqui)

**DOI:** 10.1590/S1984-29612025024

**Published:** 2025-05-23

**Authors:** Raimundo Rosemiro Jesus Baia, Carliane Maria Guimarães Alves, Marcos Sidney Brito Oliveira, Amanda Mendes Pacheco, Mosa Valdina Ferreira Moreira, Marcela Nunes Videira, Eliane Tie Oba Yoshioka, Marcos Tavares-Dias

**Affiliations:** 1 Programa de Pós-graduação em Biodiversidade Tropical – PPGBio, Universidade Federal do Amapá - UNIFAP, Macapá, AP, Brasil; 2 Universidade do Estado do Amapá - UEAP, Macapá, AP, Brasil; 3 Embrapa Amapá, Macapá, AP, Brasil

**Keywords:** Anthelmintic, Roundup, parasites, freshwater fish, treatment, Antihelmíntico, Roundup, parasitos, peixe de água doce, tratamento

## Abstract

This study investigated for the first time the antiparasitic effects of therapeutic baths with glyphosate against monogeneans of *Colossoma macropomum* (tambaqui), and alterations in the hematology and histopathology caused by the treatments. In the therapeutic baths with 250 mg/L of glyphosate for 2 hours, during 6 consecutive days, the efficacy (98.5%) against monogeneans (*Anacanthorus spathulatus*, *Mymarothecium boegeri*, and *Notozothecium janauachensis*) in the gills of this host was high. Therapeutic baths with this concentration of glyphosate caused increase in plasma glucose and total protein levels, total number of immature leukocytes, and decrease in the total number of eosinophils. Gills of fish exposed to 250 mg/L of glyphosate showed epithelial detachment, hyperplasia, and hypertrophy, resulting in partial fusion and, occasionally, complete fusion of secondary lamellae, lesions with damage ranging from moderate to severe. Although this chemotherapeutant was effective in controlling monogeneans in the gills of tambaqui and caused few physiological changes, grave histopathological changes were observed. Despite this study providing evidence for a novel chemotherapeutant to control and treat infections by monogeneans in tambaqui, the utilization of glyphosate could not be recommended due to the limitations in the tolerance of this host fish and the gill damage caused.

## Resumo

Este estudo investigou, pela primeira vez, os efeitos antiparasitários de banhos terapêuticos com glifosato contra monogenéticos nas brânquias de *Colossoma macropomum* (tambaqui) e as alterações na hematologia e histopatologia causadas pelos tratamentos. Nos banhos terapêuticos com 250 mg/L de glifosato, durante 2 horas por 6 dias consecutivos, a eficácia (98,5%) contra monogenéticos (*Anacanthorus spathulatus*, *Mymarothecium boegeri* e *Notozothecium janauachensis*) nas brânquias desse hospedeiro foi alta. Banhos terapêuticos com essa concentração de glifosato causou aumento nos níveis plasmáticos de glicose e proteínas totais, número total de leucócitos imaturos, bem como redução no total número de eosinófilos. Nas brânquias dos peixes expostos a 250 mg/L de glifosato, ocorreu descolamento do epitélio, hiperplasia e hipertrofia, que resultou na formação de fusão parcial e, ocasionalmente, houve fusão total das lamelas secundárias, lesões com danos variando de moderados a severos. Embora esse quimioterápico tenha sido eficaz no controle de monogenéticos nas brânquias de tambaqui e tenha causado poucas alterações fisiológicas, foram observadas alterações histopatológicas graves neste órgão respiratório. Apesar deste estudo ter fornecido evidências de um novo quimioterápico para controlar e tratar infecções por monogenéticos em tambaqui, o uso de glifosato não pôde ser recomendado, devido às limitações na tolerância desse peixe hospedeiro e os danos causados nas brânquias.

## Introduction

Glyphosate has been the most widely used herbicide globally due to its lo w cost and effectiveness in weed control in agriculture, and its commercialization of US$1.2 billion/year (Amarante-Junior et al., 2002; Braz-Mota et al., 2015; Bojarski et al., 2022; Moraes et al., 2023; Azadikhah et al., 2023; Paswan & Kumari 2024; Bou-Mitri et al., 2025). Glyphosate is a broad-spectrum and non-selective organophosphorus herbicide extensively applied in agricultural and aquatic systems worldwide. Its toxicity to humans and animals remains controversial, as its mode of action is the inhibition of 5-enolpyruvylshikimate-3-phosphate synthase, a pivotal enzyme of the shikimate pathway, which is absent in animals (Ma et al., 2019; Bou-Mitri et al., 2025). Therefore, glyphosate is considered relatively low toxic or non-toxic to animals and humans (Amarante-Junior et al., 2002; Ma et al., 2019; Bou-Mitri et al., 2025). Due to the worldwide application of more than 750,000 tons of glyphosate per year, this herbicide and/or its metabolites have been detected in all ecosystems of the planet (Drechsel et al., 2024). Thus, in surface waters from South America, including Brazil, glyphosate has been detected in concentrations ranging from 0.1 to 2.16 mg/L (Blasco et al., 2021). Given the extensive use of glyphosate in agriculture production, there are several routes by which aquatic organisms can be exposed to this herbicide, including fish species. Hence, with this great use of glyphosate there is a need to investigate and identify the toxicity, due to this high risk for aquatic environmental contamination (Amarante-Junior et al., 2002; Sridhar et al., 2025), as well as to identify the potential adverse effects of this exposure on fish species.

Numerous studies have reported the adverse effects of glyphosate on different animal populations, including different fish species (Annett et al., 2014; Vieira et al., 2019; Bojarski et al., 2022; Drechsel et al., 2024). It has been demonstrated that the use of glyphosate affected blood parameters, tissues of organs, behavior, and locomotion of exposed fish, leading to changes in their swimming patterns, as well as in other essential activities such as foraging, mating, and escape from predators, and irritation in the eyes, respiratory system and skin, because these animals are more sensitive to this chemical product (Jiraungkoorskul et al., 2002; Amarante-Junior et al., 2002; Ames et al., 2022; Bernardi et al., 2022; Lopes et al., 2022; Azadikhah et al., 2023; Moraes et al., 2023; Sridhar et al., 2025). For different fish species, the observed toxicity of glyphosate has been variable, depending on the species and concentration used in the exposure (Jiraungkoorskul et al., 2002; Shiogiri et al., 2012; Braz-Mota et al., 2015; Blasco et al., 2021; Sridhar et al., 2025). However, the toxicity of pure glyphosate to fish would be relatively low if its commercial formulations did not contain surfactant mixtures (Blasco et al., 2021). Fish aquaculture in different countries, including Brazil, are close to agricultural areas, and glyphosate leaching and runoff can contaminate their water supply. However, these farms also apply this herbicide to control harmful and invasive aquatic weeds such as algae and macrophytes in their culture tanks (Blasco et al., 2021; Sridhar et al., 2025).

In the global aquaculture industry, diverse chemotherapeutants are adopted to control and treat parasitic diseases, and most of them come from agriculture (e.g., emamectin benzoate, trichlorfon, parathion, diflubenzuron, teflubenzuron and copper sulfate, among others). Nevertheless, most of these products are not regulated for use in aquaculture in many countries (Busatto et al., 2018; Hoai, 2020; Rigos et al., 2024), including Brazil. Consequently, this herbicide has not been used in fish aquaculture as chemotherapeutant.

The first antiparasitic study on glyphosate was conducted by Everett & Dickerson (2003), who demonstrated that 10.1 mg/L of glyphosate had no effect *in vitro* against the protozoans *Tetrahymena thermophila* Nanney & McCoy, 1976 and 2.5 mg/L against *Ichthyophthirius multifiliis* Fouquet, 1876. Later, Kelly et al. (2010) investigated the exposure to 0.36, 3.6, and 36 mg/L of glyphosate and observed that this higher concentration affected the production and release of metacercariae of the trematode *Telogaster opisthorchis* Macfarlane, 1945 in its intermediate host, the mollusk *Potamopyrgus antipodarum* Gray, 1843. In addition, the survival of juveniles of *Galaxias anomalus* Stokell, 1959 was not influenced by the exposure to 0.36 mg/L of glyphosate, while the simultaneous exposure to this trematode and herbicide reduced the survival of these host fish. Recently, different concentrations of glyphosate were tested *in vitro* against monogeneans *Anacanthorus spathulatus* Kritsky, Thatcher & Kayton, 1979; *Mymarothecium boegeri* Cohen & Kohn, 2005, and *Notozothecium janauachensis* Belmont-Jégu, Domingues & Martins, 2004 from the gills of *Colossoma macropomum* Cuvier, 1818 (tambaqui); demonstrating a high antiparasitic efficacy. Furthermore, these studies also analyzed the tolerance of *C. macropomum* to exposure to glyphosate at concentrations of 250; 500; 1,000; 2,000; 3,000; 4,000, and 5,000 mg/L, and observed that this fish species was able to tolerate only the lowest concentration tested (Baia et al., 2024). However, glyphosate has not been used in therapeutic baths against monogeneans of fish species (Baia et al., 2024).

Dactylogyrids are ectoparasites that cause high mortality of farmed freshwater fish. These helminths have a high reproductive capacity, leading to a rapid proliferation of infections to produce a large number of parasites capable of causing mortality in host fish populations, which leads to considerable economic losses in fish aquaculture production worldwide (Tavares-Dias & Martins 2017; Grano-Maldonado et al., 2018; Hoai, 2020; Leis et al., 2023), given the histopathological effects caused mainly in the gills of host fish (Tavares-Dias et al., 2021). Hence, the management and control of these ectoparasites are crucial for the global growth of the fish aquaculture industry, posing a constant challenge for this important industry that produce food with high protein to human populations around world. Numerous chemical treatments have been employed to manage infections by monogeneans with varying degrees of success. However, a key consideration when using these chemicals is their toxicity to both the host fish and the parasites, which can vary based on biotic and abiotic conditions (Hoai, 2020; Buchmann, 2022). Tambaqui is a specie economically important for Brazilian and Amazonian fish aquaculture, because is the most farmed native species in Brazil, due to its diverse zootechnical characteristics favorable to intensive fish aquaculture (Braz-Mota et al., 2015; Tavares-Dias et al., 2021; Luz et al., 2021; Baia et al., 2024). Thus, the aim of this study was to investigate the antiparasitic effects of therapeutic baths with glyphosate and the resulting hematological and histopathological changes in *C. macropomum*, species of native fish from the Amazon and Orinoco basins.

## Material and Methods

### Fish, acclimatization and the monogenean parasite

Specimens of *C. macropomum* (± 65 g) were obtained from a commercial fish farm in the municipality of Macapá, State of Amapá, Brazil. The fish were transported to the Aquaculture Laboratory of Embrapa Amapá and acclimatized for 10 days in a 500 L tank in tanks with constant aeration and continuous water renewal, and fed *ad libitum* twice a day with a diet containing 32% crude protein (Guabi^®^, Brazil). Previously, 10 fish were examined for the presence of monogeneans and all were parasitized. These fish naturally parasitized by monogeneans were used in all assays.

Organic matter from the bottom of the tanks was siphoned every two days. The following water parameters were monitored every two days: mean temperature (30.1 ± 0.1°C), dissolved oxygen (5.7 ± 0.2 mg/L), pH (5.6 ± 0.1), total ammonia (0.4 ± 0.2 mg/L), alkalinity (10.0 ± 0.001 mg/L), and hardness (10.0 ± 0 mg/L), using a multiparameter probe (YSI, USA).

### Glyphosate composition

A commercial formulation of Roundup^®^ Original DI (Monsanto Company, São Paulo, Brazil) containing 815 g/L of glyphosate [N-(phosphonomethyl) glycine] as the active ingredient was used.

### Therapeutic baths with glyphosate against monogeneans in the gills of *C. macropomum*

For therapeutic baths, specimens of *C. macropomum* (72.8 ± 18.8 g and 15.9 ± 1.3 cm), naturally parasitized by monogeneans, were randomly distributed in 6 tanks with a capacity of 100 L of water, using two treatments and three replications of 13 fish in each replicate (39 fish per treatment). The treatments used were as follows: a control group with water from the culture tank and a group exposed to 250 mg/L of glyphosate, as previously determined in a tolerance test (Baia et al., 2024). Fish in all treatments were kept in a static water system upon adding 250 mg/L of glyphosate for 2 hours (7:00-9:00 am) of bathing for 6 consecutive days. After each daily 2 hours bath, the water in the tanks was maintained in a continuous flow system (0.003 m^3^ during 1 hour, and after with 0.0011 m^3^) for 24 hours, in the tanks with glyphosate and control treatments with water from the culture tank.

On the sixth day of therapeutic baths, the fish were euthanized by medullary section and the gills of 10 fish from each replicate per treatment (30 fish) were collected and fixed in 5% formalin for counting monogeneans, following the recommendations of Eiras et al. (2006). The prevalence and mean abundance of infection caused by monogeneans were calculated (Bush et al., 1997). The efficacy of the treatments was estimated according to a previously described methodology (Wang et al., 2008).

### Blood parameters of *C. macropomum* after therapeutic baths with glyphosate

After six 2-h therapeutic baths with glyphosate, 5 fish from each replicate (15 fish per treatment) were used to evaluate blood parameters. A blood sample was drawn from each fish by puncturing the caudal vein using syringes containing ethylenediamine tetra-acetate (10%). Whole blood was used for the following determinations: hematocrit by the microhematocrit method, total erythrocyte count in a Neubauer chamber, and hemoglobin concentration by the cyanmethemoglobin method. The Wintrobe hematimetric indices: mean corpuscular volume (MCV) and mean corpuscular hemoglobin concentration (MCHC) were calculated. Blood smears were made and panchromatically stained with a combination of May Grünwald-Giemsa-Wright combination for differential leukocyte counts in up to 200 cells of interest in each blood smear. The identification and nomenclature of the differential leukocyte counts were carried out according to recommendations of Ranzani-Paiva et al. (2013). The number of total leukocytes and thrombocytes was determined using an indirect method following previous recommendations (Ranzani-Paiva et al., 2013).

The remaining blood was centrifuged at 75 G (Centrifuga MCD-2000, Brazil) for 7 min to obtain plasma and determine glucose and total protein levels. Glucose concentration was determined by the enzymatic-colorimetric glucose oxidase method using a commercial kit (Biotécnica^®^, MG, Brazil). Total protein plasma concentration was determined by the biuret method using a commercial kit (Biotécnica^®^, MG, Brazil). These biochemical parameters were read in a UV/Visible spectrophotometer (KASVI-Model 2022/2025) using different wavelengths. Glucose levels were read in 510 nm and total protein in 550 nm.

### Histopathological analysis of *C. macropomum* gills after therapeutic baths with glyphosate

After therapeutic baths with glyphosate, gill arches from three fish from each replicate (9 fish per treatment) were collected for histopathological analysis. The first gill arch from each side (right and left) was collected and fixed in Davidson's solution for 48 hours. Then, the gill arches were dehydrated in a graded series of ethanol (70, 80, 90, 100%) and xylene, and embedded in paraffin to obtain 5 μm slices using a microtome (Easypath EP 31–20,093, Brazil). After mounting in glass slides (in duplicates), they were stained with hematoxylin and eosin (HE) and analyzed. The images were captured using a digital camera (Moticam 2300 3.0 M Pixel) coupled to a standard optical microscope and a computer. Histopathological changes were analyzed semi-quantitatively using mean assessment values (MAV) (Schwaiger et al., 1997) and histopathological alteration index (HAI) (Poleksić & Mitrović-Tutundžić, 1994).

### Statistical analysis

The histopathological, parasitological, and blood data were evaluated for normality using the Shapiro-Wilk test and the RVAideMemoire package (Herve, 2023) and homoscedasticity using the car package (Fox & Weisberg, 2019). The Mann-Whitney test was applied to compare treatments since the data did not present a normal distribution. These analyses were run using the R Core Team software (R Core Team, 2022).

## Results

### Therapeutic baths with glyphosate against monogeneans in the gills of *C. macropomum*

During the six days of exposure to 250 mg/L of glyphosate, no fish died in any of the treatments. The gills of *C. macropomum* specimens were parasitized by monogeneans *A. spathulatus*, *M. boegeri*, and *N. janauachensis*. All fish in the control group had their gills parasitized by monogeneans. There was a reduction (p<0.001) in the average abundance of monogeneans in fish exposed to 250 mg/L of glyphosate compared to fish in the control group (Table 1).

**Table 1:** Parasitological indices of monogeneans on the gills of *Colossoma macropomum* (tambaqui) subjected to therapeutic baths with glyphosate.

**Treatments**	**n**	**Prevalence (%)**	**Mean abundance**	**Efficacy (%)**
Water	30	100	27.5 ± 10.7^a^	-
250 mg/L	30	26.6	0.4 ± 0.78^b^	98.5

Data express mean ± standard deviation. Different letters, in the same column, indicate significant differences between treatments (p<0.05).

### Blood parameters of *C. macropomum* exposed to glyphosate

Fish exposed to 250 mg/L glyphosate had higher (p<0.05) plasma glucose and total protein levels compared to fish in the control group with culture tank water. The total number of eosinophils was lower (p<0.05) in fish exposed to 250 mg/L glyphosate compared to control. The number of immature leukocytes was greater (p<0.05) in fish exposed to 250 mg/L glyphosate. The total number of erythrocytes, hemoglobin, hematocrit, MCV, MCHC, total number of thrombocytes, leukocytes, lymphocytes, monocytes, and PAS-positive granular leukocytes (PAS-GL) and neutrophils did not change (p>0.05) after exposure to 250 mg/L glyphosate (Table 2).

**Table 2:** Blood parameters of *Colossoma macropomum* (tambaqui) subjected to therapeutic baths with glyphosate.

**Parameters**	**Control with water (n = 15)**	**250 mg/L (n = 15)**	**W**	**p-value**
Glucose (g/dL)	68.7 ± 13.5^a^	123.1 ± 29.7^b^	10	<0.001
Total protein (mg/dL)	2.0 ± 0.7^a^	2.8 ± 0.6^b^	51	0.011
Erythrocytes (x 10^6^ /μL)	1.3 ± 0.3^a^	1.4 ± 0.3^a^	90	0.361
Hemoglobin (g/dL	6.5 ± 0.6^a^	6.5 ± 0.9^a^	119	0.803
Hematocrit (%)	24.5 ± 2.1^a^	25.7 ± 4.2^a^	97	0.533
MCV (fL)	201.8 ± 51.6^a^	198.6 ± 57.3^a^	117.5	0.851
MCHC (g/dL)	26.8 ± 3.1^a^	25.4 ± 2.6^a^	137.5	0.309
Thrombocytes (μL)	65,522 ± 16,307^a^	55,463 ± 22,228^a^	155	0.081
Leukocytes (μL)	130,991 ± 29,499^a^	138,298 ± 32,477^a^	94	0.461
Lymphocytes (μL)	52,195 ± 25,563^a^	57,308 ± 20,511^a^	97	0.539
Monocytes (μL)	36,352 ± 14,875^a^	30,269 ± 10,461^a^	145	0.187
Neutrophils (μL)	35,356 ± 67,657^a^	34,393 ± 25,168^a^	75	0.126
Eosinophils (μL)	16,441 ± 26,611^a^	5502 ± 2859^b^	34	<0.001
PAS-GL (μL)	2169 ± 1039^a^	15,369 ± 10,437^a^	78	0.160
Immature leukocytes (μL)	120 ± 466^a^	2459 ± 2242^b^	17	<0.001

Data express mean ± standard deviation. Different letters, in the same column, indicate significant differences between treatments (p<0.05). W: Mann-Whitney test; PAS-GL: Positive-PAS granular leukocytes; MCHC: Mean corpuscular hemoglobin concentration; MCV: Mean corpuscular volume.

### Histopathology of the gills from *C. macropomum* exposed to glyphosate

**Figure 1:**
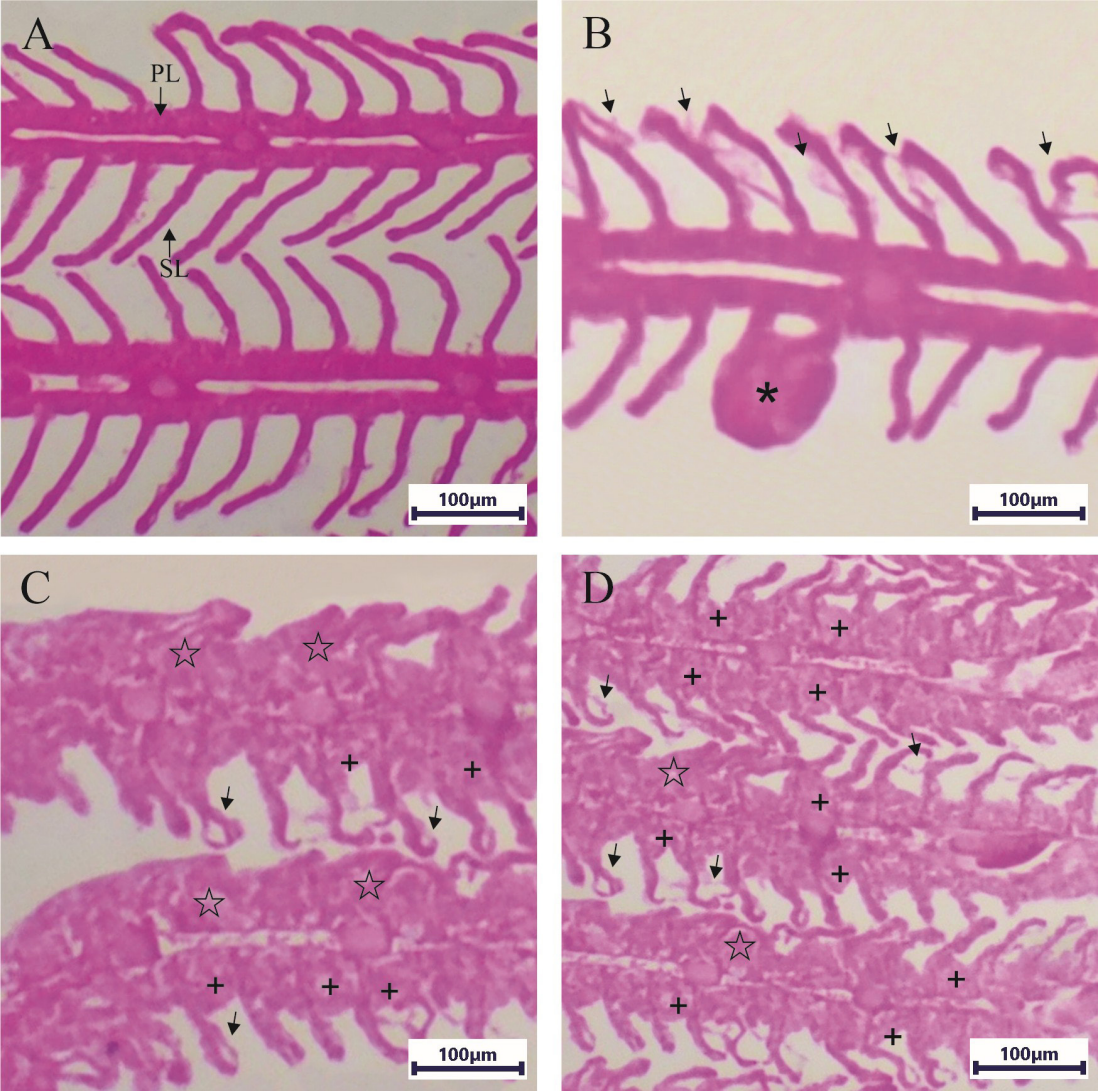
Histopathology of the gills from *Colossoma macropomum* (tambaqui) exposed to 250 mg/L of glyphosate and culture tank water. (**A**) Gills of fish exposed to culture tank water (control) showing primary (PL) and secondary (SL) lamellae. **(B**) Aneurysm (asterisks) and detachment of the lamellar epithelium (arrows) in the gills of fish exposed to culture tank water. (**C**) Total hyperplasia with fusion of the lamellae (☆); hyperplasia with partial fusion of the lamellae (+) and detachment of the lamellar epithelium (arrows) in fish exposed to 250 mg/L of glyphosate. (**D**) Hyperplasia with partial fusion of the lamellae (+) and detachment of the lamellar epithelium (arrows) in fish exposed to 250 mg/L of glyphosate.

Fish exposed to 250 mg/L glyphosate showed histopathological alterations in the gills, involving epithelial detachment, hyperplasia, and hypertrophy, resulting in partial fusion and, in some cases, complete fusion of the secondary lamellae. In the fish of the control group exposed only to water from the culture tank, histopathological alterations were less severe, including detachment of the lamellar epithelium, aneurysm, and hyperplasia with complete fusion of the lamellae (Figure 1). In the semi-quantitative evaluation, the results showed that fish exposed to 250 mg/L glyphosate presented higher values (W = 14; p = 0.02) for the histological alteration index (HIA) and mean assessment value (MAV) (W = 26.5; p = 0.211) compared to the control group exposed to culture tank water. Furthermore, mild to moderate damage was observed in the gills of control fish, while moderate to severe damage was found in fish subjected to therapeutic baths with 250 mg/L of glyphosate (Table 3).

**Table 3:** Histopathological alteration index (HAI) and mean assessment values (MAV) for the gills of *Colossoma macropomum* (tambaqui) subjected to therapeutic baths with glyphosate.

**Treatments**	**n**	**MAV**	**HAI**	**Severity of the lesions according to HAI**
Water	9	4.4 ± 2.0^a^	12.9 ± 6.3^a^	Mild to moderate organ damage
250 mg/L	9	10.3 ± 5.0^b^	15.9 ± 7.0^a^	Moderate to severe alterations in the organ

Data express mean ± standard deviation. Different letters, in the same column, indicate significant differences between treatments (p<0.05). N: Sample number.

## Discussion

Parasitic infections have a major negative impact on global aquaculture different fish species, a serious issue for fish aquaculture industry in several parts of the world (Grano-Maldonado et al., 2018; Hoai, 2020; Buchmann, 2022; Rigos et al., 2024). Therefore, control programs of various kinds are needed; however, chemotherapeutants have been the first convenient the choice of fish farmers. Consequently, different chemotherapeutants with variety toxicity are well known in aquaculture (Wang et al., 2008; Hoai, 2020; Buchmann, 2022), due to usual utilization and high resistance in the control of parasites; while others have not been yet tested. For example, parasites such as monogeneans cause major economic losses in the global fish aquaculture industry (Tavares-Dias & Martins 2017; Grano-Maldonado et al., 2018; Hoai, 2020; Leis et al., 2023), therefore posing a major challenge due to the limited availability of licensed and effective chemicals with low potential for triggering resistance when frequently used (Baia et al., 2024). Furthermore, in many situations, the choice of chemotherapy treatment strategy is not only made based on ease of application and efficacy but also on cost-effectiveness.

The present study evaluated the efficacy of 250 mg/L of glyphosate in the control of *A. spathulatus*, *M. boegeri*, and *N. janauachensis* monogeneans in the gills of *C. macropomum*, demonstrating that 2 h daily therapeutic baths were highly effective against such ectoparasites, without causing any mortality of exposed fish throughout the experimental period. Similarly, previous studies with 2,000; 3,000; 4,000, and 5,000 mg/L of glyphosate reported 100% *in vitro* efficacy against these same monogenean species (Baia et al., 2024). Therefore, these results suggest that glyphosate is an anthelmintic to control and treat infections caused by monogeneans in *C. macropomum*, despite the controversies related to its toxicity in fish exposed to this herbicide, which is unusual as chemotherapeutant in fish aquaculture. However, studies show using tissue analyses (muscle, head, dorsal spine, and caudal fin) that glyphosate (e.g., 5,600 μg/L) and its main transformation product, aminomethylphosphonic acid (AMPA) are absorbed by the body tissues (muscle, head, dorsal spine and caudal fin) of exposed fish and remain detectable even after three weeks of recovery in clean water (Drechsel et al., 2024). In *Clarias gariepinus* Burchell 1822, results of glyphosate accumulation in muscle tissue showed an increase proportional to the growing concentration tested (Ujowundu et al., 2017). In this way, as the maximum time of accumulation of the glyphosate in the muscle of fish evaluated by Drechsel et al. (2024) was only during three weeks, and indicated that for consumption of these fish this withdrawal period of was not sufficient to depuration of this herbicide; thus, this period yet needs to be determined.

In fish, blood parameters generally assess the physiological status of the population and encompass several parameters, which can be useful and easily accessible tools, in addition to reflecting rapid reactions of the organism of these animals to various environmental and non-environmental factors, as well as toxic agents and the state of the immune system. Thus, hematological analyses can facilitate the early detection of stress situations in fish farming (Bojarski et al., 2022; Witeska et al., 2023; Azadikhah et al., 2023; Ranzani-Paiva & Tavares-Dias, 2024), which can affect performance and productivity. Bojarski et al. (2022) demonstrated that hematological parameters were the most sensitive and reliable biomarkers of the exposure of carp *Cyprinus carpio* Linnaeus, 1758 to different concentrations of glyphosate. In *C. macropomum*, therapeutic baths with 250 mg/L of glyphosate increased the plasma levels of glucose and total proteins, as well as the number of immature leukocytes, reducing the total number of eosinophils. Likewise, in *C. carpio*, exposure to 5 mg/L of glyphosate also caused an increase in plasma glucose levels due to stress from exposure to this herbicide, as well as in the total number of leukocytes; but plasma levels of total proteins were not changed (Bojarski et al., 2022). In fish, glyphosate causes irritation in the eyes, respiratory system and skin (Amarante-Junior et al., 2002). However, *C. macropomum* exposed to sublethal concentrations of glyphosate (10 or 15 mg/L) did not show changes in plasma glucose levels (Braz-Mota et al., 2015). This increase in plasma levels of total proteins may be associated with changes in intracellular mechanisms and alterations in specific proteins also related to stress (Luz et al., 2021), caused by the therapeutic baths with 250 mg/L of glyphosate in *C. macropomum* of the present study. In catfish *Clarias batrachus* Linnaeus, 1758 subjected to sublethal exposure to 2.5 mg/L of glyphosate for 30 days, an increase in eosinophils was reported (Paswan & Kumari, 2024). In *C. macropomum*, this increase in the number of eosinophils and immature leukocytes may be related to the activation of the immune response due to tissue damages caused by therapeutic baths with 250 mg/L of glyphosate, influencing hematopoiesis, maturation, and release of leukocytes for bloodstream (Azadikhah et al., 2023).

The gill epithelium of fish serves as the primary interface of contact with the environment and features a large surface area, making it a significant target of chemical exposure (Braz-Mota et al., 2015; Bojarski et al., 2022; Jiraungkoorskul et al., 2002; Azadikhah et al., 2023). In the gills of *C. macropomum* exposed only to water from the culture tank, we observed detachment of the lamellar epithelium, aneurysm, and hyperplasia, resulting in the complete fusion of the lamellae. These damages ranged from mild to moderate, similar to those reported for this fish species with gills infected by *A. spathulatus*, *M. boegeri*, and *N. janauachensis* (Tavares-Dias et al., 2021). On the other hand, the gills of *C. macropomum* subjected to six baths with 250 mg/L of glyphosate showed epithelial detachment, hyperplasia, and gill hypertrophy. This treatment led to partial and, in some cases, complete fusion of the secondary lamellae, resulting in damage that varied from moderate to severe. These changes in fish gills appear to be irreversible. Nonetheless, as no investigation on recovery of this respiratory organ after exposure to glyphosate has been carried out, we suggest that such studies are needed for further information.

For grass carp *Ctenopharyngodon idella* Valenciennes, 1844 exposed to 150 ml/L of glyphosate, lamellar aneurysm, epithelial hypertrophy, basal and distal hyperplasia, leukocyte infiltration, and severe gill necrosis, accompanied by moderate lamellar fusion, were reported (Azadikhah et al., 2023). Braz-Mota et al. (2015) demonstrated that the gills of *C. macropomum* exposed to 10 or 15 mg/L of glyphosate also exhibited hyperplasia and hypertrophy of filaments, lamellar congestion, elevation of the lamellar epithelium, proliferation of mitochondria-rich cells, proliferation of mucous cells, lamellar fusion, aneurysm and rupture of lamellas; therefore, with variation of normal, moderate to severe damage, depending on the concentration tested. The gills of *C. carpio* exposed to 1 and 5 mg/L of glyphosate showed fusion of secondary lamellae, lamellar elevation, vasodilation and aneurysms of blood vessels, necrosis, proliferation, edema and fusion of epithelial cells (Bojarski et al., 2022). In general, such histopathological changes in fish gills may be compensatory responses that act on the defense mechanisms of this respiratory organ, which is also of great importance for ionic regulation in these animals (Braz-Mota et al., 2015; Jiraungkoorskul et al., 2002).

In conclusion, six therapeutic baths with concentration of 250 mg/L of glyphosate effectively controlled monogeneans in the gills of *C. macropomum*, causing minimal physiological changes. However, severe histopathological findings in the gills were also observed. Therefore, the use of glyphosate necessitates a deep understanding of environmental contamination, taking into consideration the histopathological effects caused in the fish exposed to this herbicide. Consequently, when the use of this novel chemotherapeutant for the treatment of monogenesis in fish is deemed necessary, fundamental precautions should be strictly adhered to. Due to the efficacy of this chemotherapeutant, which was responsible for mortality of parasites, further investigation is necessary to determine its mode of action. Nevertheless, combined strategies using integrated management against infections caused by monogeneans, involving adequate administration practices with limited use of chemical products, should be a priority in the fish farms to avoid monogenesis in fish populations.
